# A network pharmacology approach to predict potential targets and mechanisms of “*Ramulus Cinnamomi (cassiae) – Paeonia lactiflora*” herb pair in the treatment of chronic pain with comorbid anxiety and depression

**DOI:** 10.1080/07853890.2022.2031268

**Published:** 2022-01-31

**Authors:** Hao-Tian Pan, Zi-Qi Xi, Xu-Qiang Wei, Ke Wang

**Affiliations:** Acupuncture Anesthesia Clinical Research Institute, Yueyang Hospital of Integrated Traditional Chinese and Western Medicine, Shanghai University of Traditional Chinese Medicine, Shanghai, China

**Keywords:** “Gui Zhi–Shao Yao” herb pair, chronic pain, anxiety, depression, network pharmacology

## Abstract

**Background:**

Traditional Chinese medicine (TCM) prescriptions have multiple bioactive properties. “Gui Zhi–Shao Yao” herb pair is widely used to treat chronic pain (CP), as well as anxiety and depression. However, its related targets and underlying mechanisms have not been deciphered.

**Methods:**

In this study, the network pharmacology method was used to explore the bioactive components and targets of “Gui Zhi–Shao Yao” herb pair and further elucidate its potential biological mechanisms of action in the treatment of CP with comorbid anxiety disorder (AD) and mental depression (MD).

**Results:**

Following a series of analyses, we identified 15 active compounds, hitting 130 potential targets. After the intersections the targets of this herb pair and CP, AD and MD – sorted by the value of degree – nine targets were identified as the vital ones: Akt1, IL6, TNF, PTGS2, JUN, CASP3, MAPK8, PPARγ and NOS3. Gene ontology (GO) and Kyoto Encyclopedia of Genes and Genomes (KEGG) analysis results demonstrated 11 pathways, such as AGE-RAGE signalling pathway, IL-17 signalling pathway, TNF signalling pathway, which primarily participate in the pathological processes.

**Conclusions:**

This study preliminarily predicted and verified the pharmacological and molecular mechanisms of “Gui Zhi–Shao Yao” herb pair for treating CP with comorbid AD and MD from a holistic perspective. *In vivo* and *in vitro* experiments will be required to further investigate the mechanisms.

**KEY MESSAGE**
- A network pharmacology approach was applied to identify key targets and molecular mechanisms.
- Nine targets were regarded as the vital targets for chronic pain with comorbid anxiety and depression.
- Predicted 11 pathways were the potential therapy targets and pharmacological mechanism of “Gui Zhi–Shao Yao” herb pair.

## Introduction

Recently, the International Association for the Study of Pain (IASP) has revised the definition of pain to “an unpleasant sensory and emotional experience associated with, or resembling that associated with actual or potential tissue damage” [1]. Among various forms of pain, chronic pain (CP) accounts for 18% of total instances of pain in developing countries [2]. CP has become a global health problem, which may lead to the severe disability, with social and economic implications in the community. Negative emotions, such as anxiety and depression, are highly prevalent in patients suffering from CP [3]. The synchrony of change exists between depressive/anxiety symptoms and CP [4]. Hitherto, non-steroidal anti-inflammatory drugs (NSAIDs) – such as diclofenac, ketoprofen and naproxen – have been the first choice to treat CP as well as depression and anxiety [5]. However, the use of NSAIDs is accompanied by numerous side effects, such as gastrointestinal bleeding and ulceration, pruritus, dizziness and dysphoria [6,7]. Thus, an alternative therapy with equivalent effectiveness but fewer side effects is desperately needed for treating this comorbidity.

Traditional Chinese medicine (TCM) is a treasure of China and has formed a unique and complete theoretical system different from Western medicine [8]. Chinese herbs have widely been applied to treat diseases with precise efficacy, relatively low toxicity and low-cost [9]. *Ramulus Cinnamomi* (Gui Zhi in Chinese)–*Paeonia lactiflora* (Shao Yao in Chinese) is a classic traditional Chinese herb pair, which can reconcile the camp and guard, clear heat and relieve pain, and warm meridians and dredge collaterals [10]. According to the theory of TCM, Gui Zhi belongs to yang, which can help the Wei Qi to resist external evil, warm the meridians and dredge collaterals to relieve pain; Shao Yao belongs to yin, which can nourish Ying Qi, clear heat and alleviate pain as well. Therefore, in TCM theory, this herb pair, the “Gui Zhi–Shao Yao” herb pair can also relieve hepatic stagnation without harming yin, which is beneficial to treat depressive/anxiety symptoms. However, the related mechanisms have not been completely elucidated.

With the development of system biology, bioinformatics and high-throughput histology, the network pharmacology technology, which integrates pharmacology and information network, has attracted much more attentions. The major features of TCM (a holistic view, and treatment based on TCM syndrome differentiation) and the characteristics of Chinese herb pair (multitarget, multichannel and multilink) are consistent with the main view of the emerging concept of network pharmacology [8,11,12]. Thus, we selected the network pharmacology approach to explore the impact of the “Gui Zhi–Shao Yao” herb pair on CP with comorbid anxiety and depression, and then to clarify the underlying mechanisms.

## Materials and methods

### Chemical compounds for “Gui Zhi–Shao Yao” herb pair

The chemical compounds of each herb in this herb pair were obtained from (1) Traditional Chinese Medicine Systems Pharmacology Database and Analysis Platform [13] (TCMSP, https://tcmspw.com/tcmsp.php, version 2.3) with screening conditions of oral bioavailability (OB) ≥30%, drug-likeness (DL) ≥0.18 and half-life (HL) ≥4 h, as described before [14]; and from (2) Traditional Chinese Medicines Integrated Database [15] (TCMID, http://www.megabionet.org/tcmid/) through SwissADME [16] (http://www.swissadme.ch/). After deleting the duplicate data, there were 26 herbal compounds of Gui Zhi and 22 herbal compounds of Shao Yao (Table S1).

### Compound targets of “Gui Zhi–Shao Yao” herb pair

We input all the active ingredients into PubChem (https://pubchem.ncbi.nlm.nih.gov/) [17] – the world’s largest collection of freely accessible chemical information – to obtain the 2  Structure or Canonical SMILES of these ingredients. Afterward, these data were imported into Swiss Target Prediction (http://www.swisstargetprediction.ch/) [18] and TCMSP database to predict the potential targets genes with the species limited as “*Homo sapiens*”. We took the intersections of the above two difference analyses, yielding a total of 130 targets genes. Then, the National Centre for Biotechnology Information’s (NCBI) Gene database (www.ncbi.nlm.nih.gov/gene) [19] was used to standardize gene names and organisms under the condition of “*Homo sapiens*” (Table S2).

### Targets of CP, anxiety and depression

The gene targets associated with diseases were collected from DisGeNET (https://www.disgenet.org/, version 7.0) [20], a discovery platform containing one of the largest publicly available collections of genes and variants associated with human diseases, and GeneCards (https://www.genecards.org/, version 5.0) [21], a searchable, integrative database providing comprehensive, user-friendly information on all annotated and predicted human genes. We screened these two platforms using the keyword “chronic pain”, “anxiety disorder (AD)” and “mental depression (MD)”, then took the part that is higher than the average of the score, and deleted the duplicate results. Eventually, we gathered 2941, 2662 and 2405 targets related to CP, AD and MD, respectively.

### Protein–protein interaction (PPI)

We utilized an online tool, jvenn (http://jvenn.toulouse.inra.fr/app/example.html) [22], to take the intersections between the targets of “Gui Zhi–Shao Yao” herb pair and diseases. Afterward, candidate targets were imported into the STRING (https://string-db.org/, version 11.0) [23] – a database of known and predicted PPIs. We selected “*Homo sapiens*” and a medium confidence score with correlation degree ≥0.400 as the cut-off value. PowerPoint (Microsoft Office 2019, Redmond, WA) was utilized to render the results.

### Gene ontology (GO) and Kyoto Encyclopedia of Genes and Genomes (KEGG) enrichment analysis

The Metascape (https://metascape.org/) [24] was used to conduct the GO and KEGG enrichment analysis (*p* value cut-off, .01). The bar chart of GO enrichment analysis and bubble chart of KEGG pathway chart were visualized by http://www.bioinformatics.com.cn, an online platform for data analysis and visualization.

### Network construction

Network construction was performed using the network visualization software Cytoscape (version 3.7.2) as follows: (1) compound–compound target network, which connects the chemical compounds of this herb pair and the related targets; (2) “Gui Zhi–Shao Yao” herb pair–diseases PPI network obtained from the STRING; (3) targets–disease–KEGG pathway network.

## Results

### Compound–compound target network analysis

**Figure 1.**
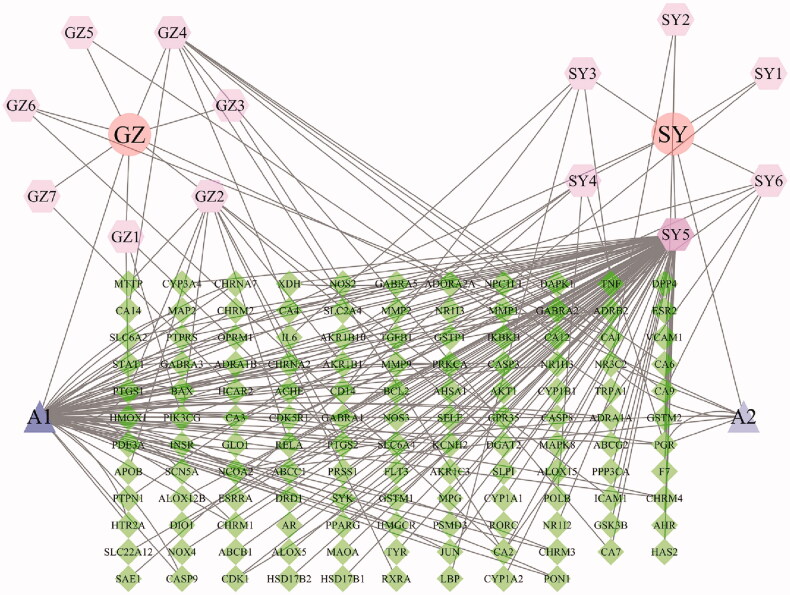
Compound–compound target network **(**diamonds represent compound targets, cycles represent the herbs, hexagons represent the compounds, triangles represent the duplicate compound of these two herbs). GZ1: (–)-taxifolin; GZ2: taxifolin; GZ3: peroxyergosterol; GZ4: 3,4-dihydroxy benzoic acid; GZ5: cinnamaldehyde; GZ6: coumarinic acid; GZ7: trans-cinnamic acid; SY1: paeoniflorigenone; SY2: (3S,5R,8R,9R,10S,14S)-3,17-dihydroxy-4,4,8,10,14-pentamethyl-2,3,5,6,7,9-hexahydro-1H-cyclopenta[a]phenanthrene-15,16-dione; SY3: paeoniflorin; SY4: mairin; SY5, kaempferol; SY6: phenol; A1: beta-sitosterol; A2: sitosterol; GZ: Gui Zhi; SY: Shao Yao.

A total of 130 predicted targets were identified from 15 candidate compounds of the “Gui Zhi–Shao Yao” herb pair (Figure 1); the other candidate compounds did not have corresponding targets. Many targets were hit by multiple compounds. For instance, PTGS1, PTGS2 and PIK3CG were modulated by multiple ingredients including (–)-taxifolin, beta-sitosterol, taxifolin and kaempferol. Other targets, such as PGR or NCOA2, also matched more than one ingredient. These data suggested that “Gui Zhi–Shao Yao” herb pair has the multi-component, multi-target and multi-disease treatment characteristics.

### Herb pair–diseases PPI network analysis

**Figure 2.**
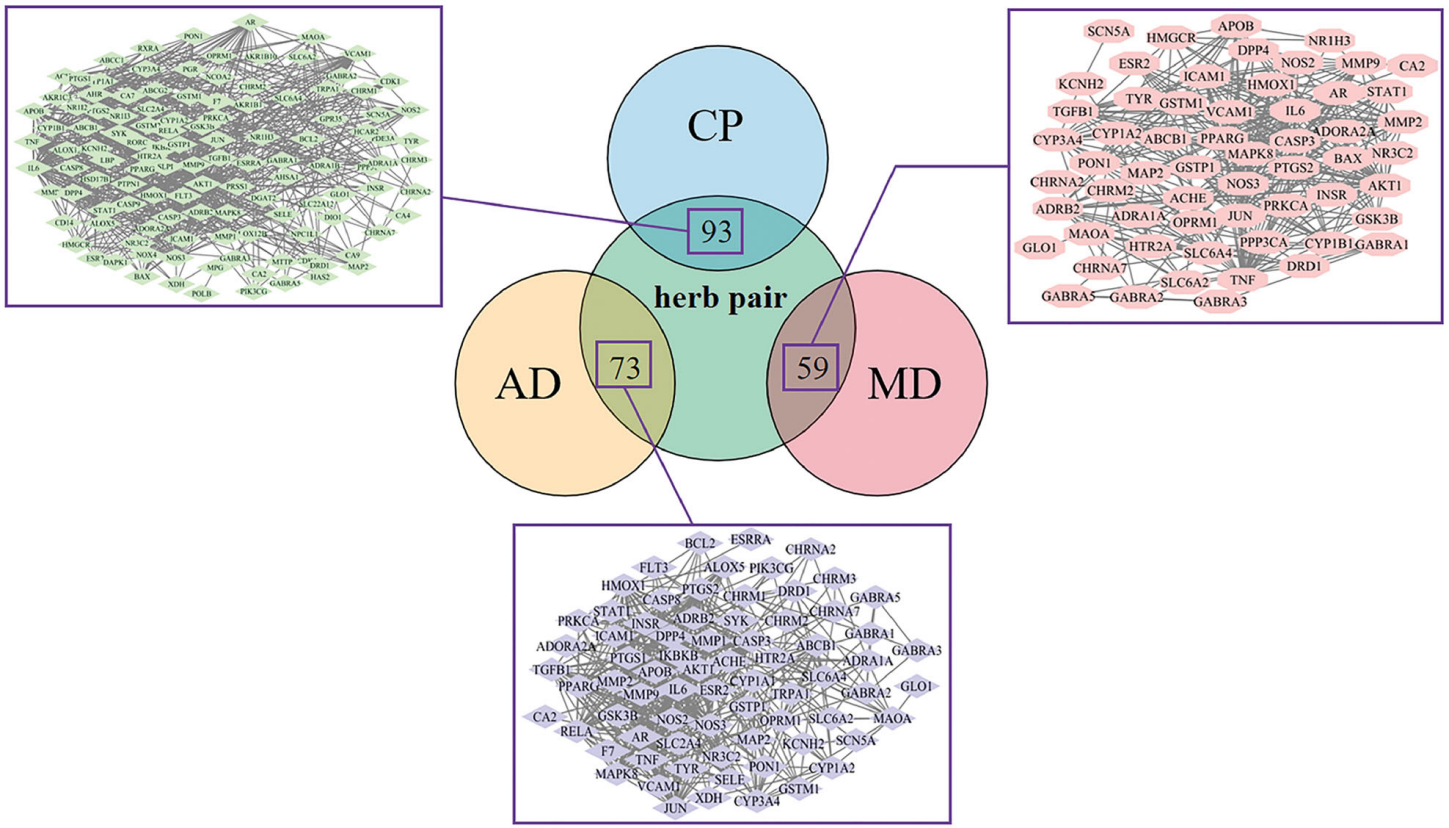
Venn of intersections and PPI network of herb pair and diseases. CP: chronic pain; AD: anxiety disorder; MD: mental depression.

The Venn diagram of the common targets between “Gui Zhi–Shao Yao” herb pair and disease and the PPI network of these common targets are shown in Figure 2. The disease-targets and the “Gui Zhi–Shao Yao” herb pair-targets were intersected. The 93, 73 and 59 common targets were found between herb pair-targets and the targets of CP, AD and MD, respectively. According to the PPI network, the top 10 highest degree targets in each intersection were selected. There were nine targets that were identical in these targets, and were regarded as key targets that play an important part in the underlying mechanisms of “Gui Zhi–Shao Yao” herb pair: Akt1, IL6, TNF, PTGS2, JUN, CASP3, MAPK8, PPARγ and NOS3. The details are displayed in Table S3.

### GO and KEGG enrichment analysis

**Figure 3.**
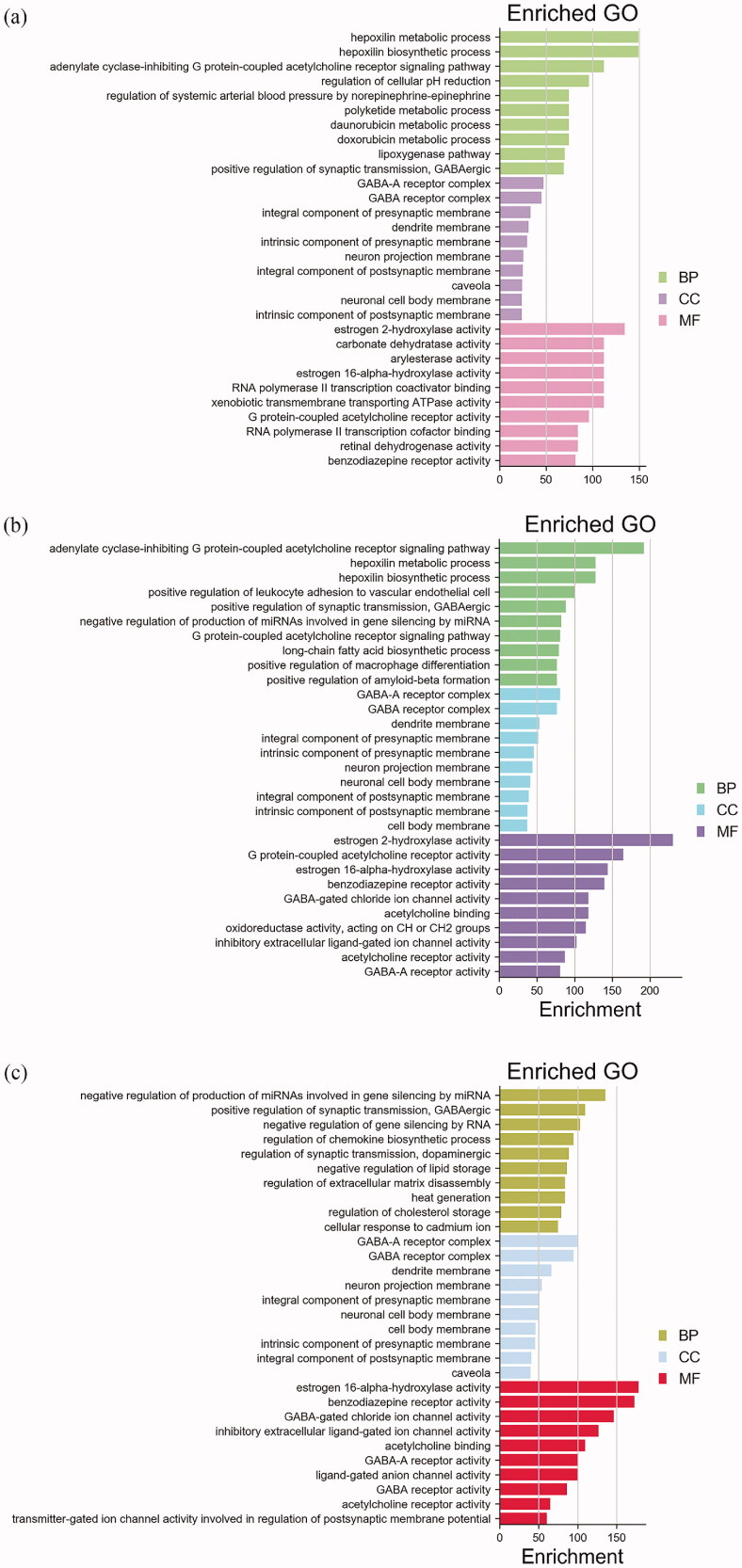
Results of GO enrichment for (a) “Gui Zhi–Shao Yao” herb pair and CP, (b) “Gui Zhi–Shao Yao” herb pair and (c) “Gui Zhi–Shao Yao” herb pair and MD. BP: biological processes; CC: cellular components; MF: molecular functions.

The GO and KEGG enrichment analysis was conducted to systematically discern the multiple therapeutic mechanisms of “Gui Zhi–Shao Yao” herb pair for CP with comorbid AD and MD. Sorted based on the value of “Enrichment”, we selected the top 10 of biological processes (BP), cellular components (CC) and molecular functions (MF), separately, as the pivotal results of GO enrichment (as shown in Figure 3). In terms of CP, the BP results suggested that these targets provided responses to small molecule metabolic process, neurotransmitter (acetylcholine, norepinephrine–epinephrine) and synaptic transmission (GABAergic); the CC results suggested that these targets mainly localized at neuronal synapses; the MF results suggested that these targets were mostly involved in receptor activity and enzyme activity (Table S4). In terms of AD, the BP results suggested that these targets were related to neurotransmitter (acetylcholine, norepinephrine–epinephrine) and synaptic transmission (GABAergic), molecule biosynthetic and metabolic process, and macrophage differentiation; the CC results suggested that these targets mainly localized at neuronal membrane; the MF results suggested that these targets were also mostly involved in regulation of receptor activity, enzyme activity, ion channel activity (Table S5). In terms of MD, the BP results suggested that these targets were related to synaptic transmission (GABAergic and dopaminergic), chemokine biosynthetic process, lipid storage, etc. the CC results suggested that these targets mainly localized at neuronal membrane; the MF results suggested that these targets were similar mostly involved in regulation of receptor activity, enzyme activity and ion channel activity (Table S6).

**Figure 4.**
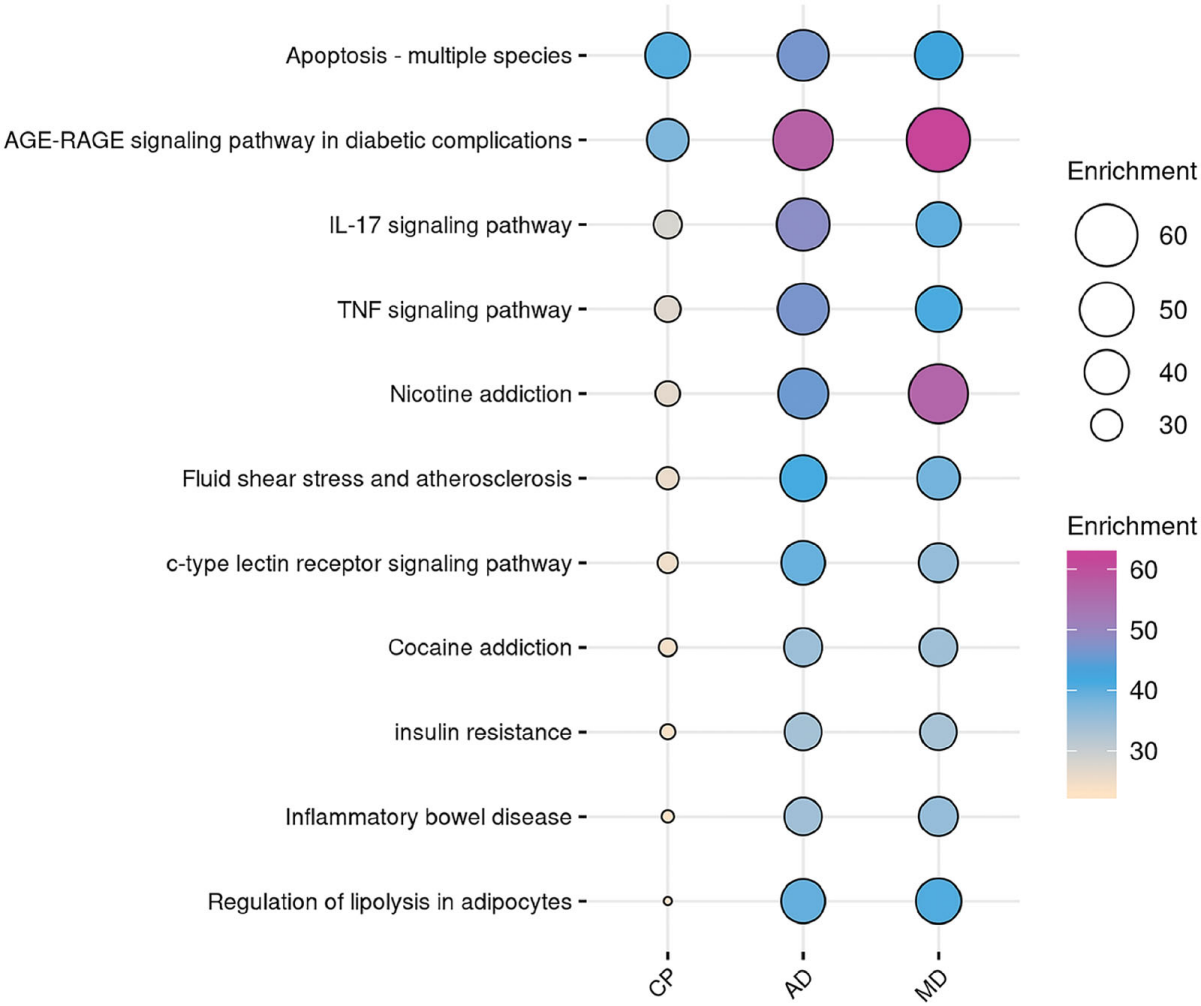
The 11 common pathways in three KEGG analysis results. CP: chronic pain; AD: anxiety disorder; MD: mental depression.

**Figure 5.**
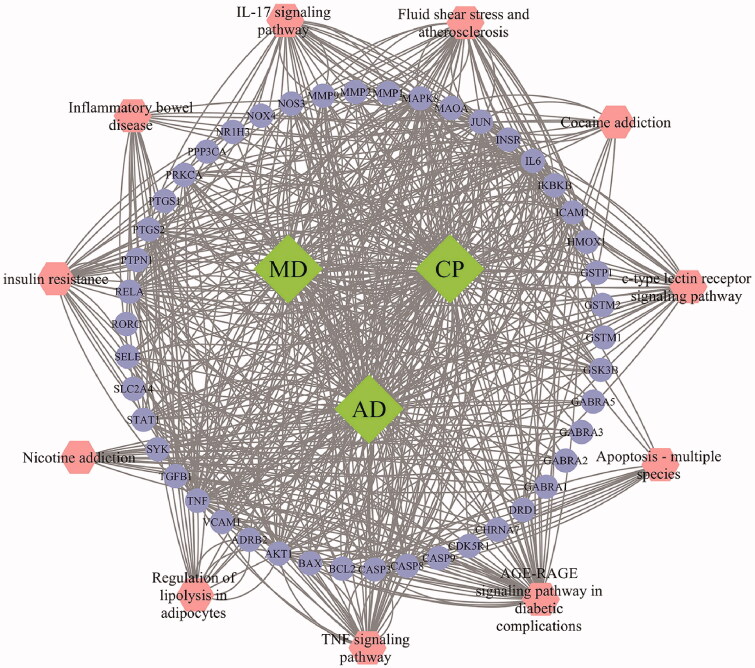
Targets–disease–KEGG pathway network **(**diamonds represent three diseases, cycles represent the targets, hexagons represent the pathways). CP: chronic pain; AD: anxiety disorder; MD: mental disorder.

As displayed in Figure 4, 11 common KEGG pathways have been screened, also sorted by the “Enrichment”. The vital underlying pathways that exist repeatedly in three KEGG analysis results are: apoptosis – multiple species, AGE-RAGE signalling pathway in diabetic complications, IL-17 signalling pathway, TNF signalling pathway, nicotine addiction, fluid shear stress and atherosclerosis, C-type lectin receptor (CLR) signalling pathway, cocaine addiction, insulin resistance, inflammatory bowel disease (IBD) and regulation of lipolysis in adipocytes, which can be summed up as three aspects: inflammatory response, immune regulation and regulation of neurotransmission. Meanwhile, the targets–disease–KEGG pathway network was constructed to visualize these results (Figure 5).

## Discussion

In this study, we predicted the active ingredients and potential targets of Gui Zhi–Shao Yao herb pair related to CP through the network pharmacology approach and we found that the potential mechanism of Gui Zhi–Shao Yao herb pair for treating CP was predominantly related to inflammatory response, immune regulation and regulation of neurotransmission. Mutual influence exists between CP and AD/MD [25,26]. They would exert long-term negative effects on pain ratings even after relief of AD or MD [4]. With chronic and persistent pain, systemic inflammation has been observed in individuals with AD/MD [27,28], which may further aggravate the conditions. Thus, CP is a multidimensional disease, mainly including sensory and affective dimension. In recent years, studies have shown a significant overlap in the neurobiological mechanisms between CP and AD/MD [25,26]. Clinically, CP can induce AD and MD, and the comorbid condition of CP associated with AD/MD usually occurs. However, persistent and even severe AD/MD may conversely affect the recovery from CP [29,30]. During the COVID-19 pandemic, the mutual influence was also observed [31]. There are extensive overlapping brain regions involved in CP, AD and MD, including hippocampus, prefrontal cortex (PFC), insular cortex, anterior cingulate, thalamus and amygdala [26]. For example, the theta-frequency power in the medial PFC and theta-frequency synchronization between the medial PFC and ventral hippocampus were significantly greater increased when CP rat was displaying elevated anxiety-like behaviours [32]. Furthermore, non-invasive, repetitive direct anodal current transcranial stimulation of the PFC could reverse established allodynia and suppressed aversion and anxiety-related behaviours of CP mice [33]. Accumulating evidence suggests that the excess of inflammatory response in the hippocampus involved in the progression of both posttraumatic stress disorder and CP [34]. “Gui Zhi–Shao Yao” herb pair has been used to reduce CP and relieve AD/MD simultaneously for a long time. Herein, under the theoretical guidance of TCM, we utilized the network pharmacology approach to explore the underlying targets and potential mechanisms of “Gui Zhi–Shao Yao” herb pair to treat CP with comorbid AD and MD.

After the analyses, nine targets were identified as critical ones involved in the therapeutic effects of “Gui Zhi–Shao Yao” herb pair on CP with comorbid AD and MD. Akt1, an important downstream substrate in the phosphatidylinositol 3-kinase (PI3K) pathway, is involved in nociceptive information processing, anxiety and depression-like behaviours. For example, intrathecal injection of Akt inhibitor MK-2206 or PI3K inhibitor LY294002 significantly attenuated mechanical allodynia and thermal hyperalgesia induced by paclitaxel [35]. AKT1 affects anxiety-like behaviour in a sex-specific fashion, which the male Akt1 KO mice increased anxiety-like behaviour [36]. The activation of the PIK3CA-AKT1 signalling pathway exerted antidepressant-like effects in the olfactory bulbectomized rat model of depression [37]. IL-6, a proinflammatory cytokine, has various effects on the nervous system, involving neuroprotection, nerve regeneration and enhancement of nociception [38]. IL-6 can induce the dorsal root ganglion nociceptor excitability and interact with IL-6R to stimulate inflammatory processes [39]. IL-6 is also strongly and consistently associated with depression and anxiety [40]. A recent study has regarded serum IL-6 as a potential predictor of the antidepressant effects of ketamine [41]. TNF is also a proinflammatory cytokine involved in several cellular responses, such as apoptosis and proliferation; recently, it has been regarded as a target for neuropathic pain [42,43]. TNF in brain has been a specific target to alleviate thermal hyperalgesia and positively influence the affective component of pain [44]. However, during animal experiments, Del Rivero et al. [45] found that only male mice responded to such a method of analgesia. There are significant changes in the levels of TNF in cells, plasma and serum in patients with AD/MD [46], which is consistent with abundant evidence that supports the role of inflammation in the development of psychological distress [47,48]. PTGS2, also named COX-2, a proinflammatory mediator, has been demonstrated to induce hypersensitization of pain transmitting neurons [49]. COX-2 is one of the two main isoforms of COX enzymes, which play a key role in the mechanism of action of NSAIDs [50]. The inhibition of COX-2 can significantly alleviate chronic mechanical allodynia [51,52]. The COX2 highly selective inhibitor lumiracoxib can prevent acute stress-induced increase in BLA cellular activity and anxiety-like behaviour in mice and reverse chronic CORT-induced increases in amygdala glutamatergic signalling and anxiety-like behaviours in rats [53,54]. In MD, the expression of COX2 was increased in the hippocampal dentate gyrus in depressed rats, and COX-2 inhibition by celecoxib significantly ameliorates depressive behaviours [55]. As one of substrates of c-Jun N-terminal kinase (JNK), JUN is an important nuclear transcription factor [56]. MAPK8 is known as JNK1. JNK1/c-Jun signalling is important in the pathogenesis of CP [57,58]. JNK also plays an important role in the development of depression via several physiological processes, such as inflammation, oxidative stress, cell death and neurogenesis [59]. For example, intracerebral ventricular infusion with a JNK inhibitor DJNKI-1 in mice can promote adult hippocampal neurogenesis in the hippocampus to alleviate anxiety [60]. Moreover, treatment with JNK inhibitor SP600125 can decrease the neuroinflammation response in the habenula, amygdala and medial PFC to alleviate depressive-like behaviours in rats [61]. Casp3, an effector caspase of apoptosis in the anterior cingulate cortex [62], can be downregulated by nerve injury. Conversely, overexpression of Casp3 reduces peripheral hypersensitivity [63]. Meanwhile, the overexpression of Casp3 in the hippocampus is an important step in the pathogenesis of depression [64]. There is evidence to support that electroacupuncture intervention could reduce the expression of caspase-3 in cortex and improve depressive symptoms as well [65]. It has also been scanned as one potential target of the herb pair of prepared Rehmannia root-Chinese arborvitae kernel for ADs [66]. PPARγ expresses primarily in neurons and some studies have shown that PPARγ activation is implicated in a decrease of specific types of neuropathic and inflammatory pain [67,68]. PPARγ agonist pioglitazone can dose-dependently inhibit the spinal glial and stimulus-evoked p-ERK activation and block the development of and reduce established neuropathic pain in rats [69]. Long-term treatment with PPARγ agonist can relieve anxiety- and depression-like symptoms through decreasing the expression of inflammatory gene programs [70]. Another study also pointed that the antidepressant- and anxiolytic-like effects produced by activation of PPARγ may be via an adiponectin-dependent mechanism [71]. NOS3, one of the three components of nitric oxide (NO), is involved in regulating several cellular processes, such as pathological pain [72]. NO signalling pathway has also been shown to play a crucial role in anxiety and depression [73,74]. The nitric oxide synthase (NOS) and concentrations of NO metabolites are higher in depressed patients [75,76]. The inhibition of NOS3 in the dorsolateral periaqueductal grey may exert anxiolytic effects [77].

The KEGG database has been developed to understand the conservation and variation of genes and genomes at the level of cellular organisms [78]. In this study, we listed 20 pathways related to CP, AD and MD (details are shown in Tables S7–S9). Among these, 11 pathways were considered the vital mechanisms involved in all of these three diseases. Neuronal apoptosis is a significant contributor to the development of hyperalgesia and sensitization particularly in neuropathic pain [79], and it is related to apoptosis-associated proteins such as caspases [80]. CP is associated with chronic neuroinflammation, the local inflammation in the peripheral or central nervous system [81]. Persistent chronic inflammation also increases the development of neurodegenerative diseases [82]. As an essential mechanism leading to neurodegeneration, apoptosis is also implicated [79] in the pathogenesis of neuropsychiatric diseases, such as anxiety and depression [83]. Advanced glycation end products (AGEs) interact with the receptor for AGEs (RAGE), contributing to an inflammatory and oxidative response [84]. AGE–RAGE pro-inflammatory signalling pathway can lead to disease pathogenesis since its activation, and ultimately tissue damage [85]. RAGE signalling is involved in the occurrence of depressive-like behaviours in rats [86]. The normalization of AGE/RAGE in the PFC and hippocampus has been found to exert antidepressant-like and anxiolytic effects [87]. IL-17 is mainly produced by immune cells and has potent proinflammatory properties [88]. It contributes to the generation of mechanical hyperalgesia [89]. IL-7 may play an important role in mediating anxiety in patients with chronic inflammatory conditions [90]. The anxiety scores correlated negatively with IL-7 [91]. IL-7 is also important in the development of depressive disorders [92,93]. TNF is a vital signalling molecule in the central nervous system in physiological and pathophysiological conditions [94]. TNF release triggers a complex downstream cascade involving the release of other cytokines and then contributes to the initiation of neuropathic and inflammatory pain [95,96]. In line with the inflammatory hypothesis of depression, suggesting that immune hyperactivation and dysregulated cytokine production are involved in depression, Zou et al. found that changes in the levels of cytokines (such as IL-1β, TNF-α and IL-8) were related to the degree of depression [97,98]. Deletion of TNF receptor 2 could show the ability to increase anxiety-like behaviour [99]. The pain-inhibitory effect of nicotine was mostly studied in animals, and it is related to the activation of nicotinic acetylcholine receptors. However, this antinociceptive effect is consistent with the chronic exposure tolerance [100]. Nicotine influences a large number of physiological processes, including AD and MD [101,102]. For example, nicotine can increase brain dopamine levels to display antidepressant effect [103]; however, depression is also a risk factor for nicotine dependence [102]. C-type lectin receptor is involved in the regulation of inflammation [104]. Macrophage-inducible C-type lectin (Mincle), one of the CLRs, is a pattern-recognition receptor (PRR) allocated to the CLR family; PRRs are regarded as molecules that induce pathological changes in CP [105]. Mincle in the injured nerve has also been demonstrated to induce neuropathic pain [106]. Administration of cocaine can relieve depression and anxiety induced by CP, accompanied by a downregulation of 5-HT1A receptor [107]. However, continuous and abusive intake of cocaine may lead to AD and MD [108,109]. CP and abnormalities in glucose metabolism have a strong relationship, which means that CP may accelerate the progression of insulin resistance. The underlying mechanisms partly correlated with downregulated expression of insulin receptors [110]. By contrast, insulin resistance can conversely promote nociceptive hypersensitivity in a hyperglycaemia-independent way [111]. Insulin resistance in brain induces mitochondrial and dopaminergic dysfunction, contributing to anxiety and depressive-like behaviours [112]. In other words, a high rate of comorbidity exists between insulin resistance and AD/MD [113,114]. Meanwhile, increased inflammation and cytokine production have been found in the insulin resistance states in some brain regions [115], which provides additional evidence for the relationship between insulin resistance and AD/MD. Chronic abdominal pain is a common symptom in IBD [116]. Related aetiology involves peripheral inflammation, which can result in the release of cytokines leading to visceral hypersensitivity, and central mechanisms, which also influence pain modulation in IBD [117,118]. Numerous psychosocial factors, including AD and MD, are positively associated with pain in IBD [119]. Moreover, there is a significant link between AD/MD and IBD itself, because proinflammatory mediators present in IBD may contribute to AD and MD [120–122]. Adipocytes linked with primary afferent neurons may participate in the development of neuropathic pain [123]. By contrast, pain may be important for the regulation of lipolysis [124]. Adipocytes can continually and systemically release proinflammatory factors [125], which suggest a relationship with AD/MD. Secreted from adipocytes, adiponectin and leptin have been demonstrated to modulate anxiety and depressive behaviours [126,127].

There have been some reports about the effects of Gui Zhi or Shao Yao on these targets. For example, Gui Zhi has the anti-inflammatory effect of down-regulating the expression of various genes related to inflammatory responses in lipopolysaccharide (LPS)-stimulated BV-2 microglial cells, including IL6, TNF and COX-2 [128]. Paeoniflorin, extracted from the root of Shao Yao, significantly decreased the expressions of p-Akt (Ser 473) in rats with collagen-induced arthritis. Paeoniflorin also could inhibited LPS-induced expression of IL6, TNF and COX-2 [129]. Total glucosides of Shao Yao could inhibit the neuronal apoptotic death by reduced CASP3 and Bax expression, and elevated Bcl-2 [130]. However, the predicted crucial pathways of the ingredients of the “Gui Zhi–Shao Yao” herb pair are very sparse by reviewing the literature, which supply new insight into possible explored the underlying mechanism of the potential anti-CP effects by “Gui Zhi–Shao Yao” herb pair.

Our study has several limitations. First, during the decoction process, various chemical components of different herbs may interact, causing certain changes in the composition. It is very likely that the chemical compositions are differences between the herbal compounds of Gui Zhi, Shao Yao and the “Gui Zhi–Shao Yao” herb pair. Second, the further experimental validation is essential to reveal these targets and pathways in anti-CP effect of the “Gui Zhi–Shao Yao” herb pair. Third, our study was based on the currently available scientific evidence. Some druggability of the compounds of “Gui Zhi–Shao Yao” are not well elucidated at present. We cannot completely exclude this possible information bias.

## Conclusions

In this study, using the network pharmacology, our study has predicted the targets of the ingredients of the “Gui Zhi–Shao Yao” herb pair and explored the underlying mechanism of the potential anti-CP effects. The extensive analysis results showed that “Gui Zhi–Shao Yao” herb pair elicits its pharmacological effects in CP by modulating the multiple pathways and multiple targets.

## Supplementary Material

Supplemental Material

## Data Availability

The data are available for reproduction of results on request from the corresponding author.
