# Supplementary material for: A network pharmacology approach to predict potential targets and mechanisms of “Ramulus Cinnamomi (cassiae) – Paeonia lactiflora” herb pair in the treatment of chronic pain with comorbid anxiety and depression
Source: Ann Med. 2022 Jan 31;54(1):413–25. doi: 10.1080/07853890.2022.2031268 (PMC8812742; doi:10.1080/07853890.2022.2031268)
Supplement: Supplemental Material [file IANN_A_2031268_SM8833.zip › Supplemental files/Table S1.docx]

**Supplementary Table S1 Compounds database of “Gui Zhi – Shao Yao” herb pair**

| Compound | herb | OB (%) | DL | HL | Lipinski violations* | Ghose violations* | Veber violations* | Egan violations* | Muegge violations* | Database |
| --- | --- | --- | --- | --- | --- | --- | --- | --- | --- | --- |
| (-)-taxifolin | Gui Zhi | 60.51 | 0.27 | 14.37 |  |  |  |  |  | TCMSP |
| beta-sitosterol | Gui Zhi, Shao Yao | 36.91 | 0.75 | 5.36 |  |  |  |  |  | TCMSP |
| sitosterol | Gui Zhi, Shao Yao | 36.91 | 0.75 | 5.37 |  |  |  |  |  | TCMSP |
| taxifolin | Gui Zhi | 57.84 | 0.27 | 14.41 |  |  |  |  |  | TCMSP |
| Peroxyergosterol | Gui Zhi | 44.39 | 0.82 | 4.06 |  |  |  |  |  | TCMSP |
| 2'-hydroxycinnamaldehyde | Gui Zhi |  |  |  | YES | NO | YES | YES | NO | TCMID |
| 2-methoxycinnamaldehyde | Gui Zhi |  |  |  | YES | YES | YES | YES | NO | TCMID |
| 3,4-dihydroxybenzoicacid | Gui Zhi |  |  |  | YES | NO | YES | YES | NO | TCMID |
| acetic acid | Gui Zhi, Shao Yao |  |  |  | YES | NO | YES | YES | NO | TCMID |
| anethole | Gui Zhi |  |  |  | YES | NO | YES | YES | NO | TCMID |
| anhydrocinnzeylanine | Gui Zhi |  |  |  | YES | YES | YES | YES | YES | TCMID |
| anhydrocinnzeylanol | Gui Zhi |  |  |  | YES | YES | YES | YES | YES | TCMID |
| benzaldehyde | Gui Zhi |  |  |  | YES | NO | YES | YES | NO | TCMID |
| camphor | Gui Zhi |  |  |  | YES | NO | YES | YES | NO | TCMID |
| cinnamaldehyde | Gui Zhi |  |  |  | YES | NO | YES | YES | NO | TCMID |
| coumarin | Gui Zhi |  |  |  | YES | NO | YES | YES | NO | TCMID |
| coumarinic acid | Gui Zhi |  |  |  | YES | YES | YES | YES | NO | TCMID |
| dihydromelilotoside | Gui Zhi |  |  |  | YES | NO | YES | NO | YES | TCMID |
| farnesol | Gui Zhi |  |  |  | YES | YES | YES | YES | NO | TCMID |
| meliloticacid | Gui Zhi |  |  |  | YES | YES | YES | YES | NO | TCMID |
| melilotocarpan a | Gui Zhi |  |  |  | YES | YES | YES | YES | YES | TCMID |
| nerolidol | Gui Zhi |  |  |  | YES | YES | YES | YES | NO | TCMID |
| procurcumenol | Gui Zhi |  |  |  | YES | YES | YES | YES | YES | TCMID |
| styrene | Gui Zhi |  |  |  | YES | NO | YES | YES | NO | TCMID |
| tetradecanal | Gui Zhi |  |  |  | YES | YES | NO | YES | NO | TCMID |
| trans-cinnamic acid | Gui Zhi |  |  |  | YES | NO | YES | YES | NO | TCMID |
| paeoniflorigenone | Shao Yao | 87.59 | 0.37 | 7.45 | YES | YES | YES | YES | YES | TCMSP, TCMID |
| (3S,5R,8R,9R,10S,14S)-3,17-dihydroxy-4,4,8,10,14-pentamethyl-2,3,5,6,7,9-hexahydro-1H-cyclopenta[a]phenanthrene-15,16-dione | Shao Yao | 43.56 | 0.53 | 4.34 |  |  |  |  |  | TCMSP |
| Lactiflorin | Shao Yao | 49.12 | 0.8 | 7.26 |  |  |  |  |  | TCMSP |
| paeoniflorin | Shao Yao | 53.87 | 0.79 | 13.88 |  |  |  |  |  | TCMSP |
| paeoniflorin_qt | Shao Yao | 68.18 | 0.4 | 8.81 |  |  |  |  |  | TCMSP |
| albiflorin_qt | Shao Yao | 66.64 | 0.33 | 6.54 |  |  |  |  |  | TCMSP |
| Mairin | Shao Yao | 55.38 | 0.78 | 8.87 |  |  |  |  |  | TCMSP |
| kaempferol | Shao Yao | 41.88 | 0.24 | 14.74 |  |  |  |  |  | TCMSP |
| (+)-catechin | Shao Yao |  |  |  | YES | YES | YES | YES | YES | TCMID |
| benzoic acid | Shao Yao |  |  |  | YES | NO | YES | YES | NO | TCMID |
| epigallocatechin | Shao Yao |  |  |  | YES | YES | YES | YES | NO | TCMID |
| paeonilactone a | Shao Yao |  |  |  | YES | YES | YES | YES | NO | TCMID |
| paeonilactone b | Shao Yao |  |  |  | YES | YES | YES | YES | NO | TCMID |
| paeonilactone c | Shao Yao |  |  |  | YES | YES | YES | YES | YES | TCMID |
| paeonol | Shao Yao |  |  |  | YES | YES | YES | YES | NO | TCMID |
| palbinone | Shao Yao |  |  |  | YES | YES | YES | YES | YES | TCMID |
| phenol | Shao Yao |  |  |  | YES | NO | YES | YES | NO | TCMID |
| pyrethrin i | Shao Yao |  |  |  | YES | YES | YES | YES | NO | TCMID |
| pyrethrin ii | Shao Yao |  |  |  | YES | YES | YES | YES | YES | TCMID |

OB, oral bioavailability; DL, drug-likeness; HL, half-life. * The Lipinski violations, Ghose violations, Veber violations, Egan violations, and Muegge violations represent five different rule-based filters to assess the possibility of a molecule becoming an oral drug from the perspective of bioavailability.
