# Supplementary material for: A network pharmacology approach to predict potential targets and mechanisms of “Ramulus Cinnamomi (cassiae) – Paeonia lactiflora” herb pair in the treatment of chronic pain with comorbid anxiety and depression
Source: Ann Med. 2022 Jan 31;54(1):413–25. doi: 10.1080/07853890.2022.2031268 (PMC8812742; doi:10.1080/07853890.2022.2031268)
Supplement: Supplemental Material [file IANN_A_2031268_SM8833.zip › Supplemental files/Table S2.docx]

**Supplementary Table S2 Targets and Official Symbol of the Ingredients**

| Target | Official Symbol | Ingredient |
| --- | --- | --- |
| Prostaglandin G/H synthase 1 | PTGS1 | (-)-taxifolin, beta-sitosterol, taxifolin, kaempferol |
| Prostaglandin G/H synthase 2 | PTGS2 | (-)-taxifolin, beta-sitosterol, taxifolin, kaempferol |
| Phosphatidylinositol-4,5-bisphosphate 3-kinase catalytic subunit, gamma isoform | PIK3CG | (-)-taxifolin, beta-sitosterol, taxifolin, kaempferol |
| Progesterone receptor | PGR | beta-sitosterol, sitosterol, (3S,5R,8R,9R,10S,14S)-3,17-dihydroxy-4,4,8,10,14-pentamethyl-2,3,5,6,7,9-hexahydro-1H-cyclopenta[a]phenanthrene-15,16-dione, Mairin, kaempferol |
| Nuclear receptor coactivator 2 | NCOA2 | beta-sitosterol, sitosterol, kaempferol |
| Muscarinic acetylcholine receptor M1 | CHRM1 | beta-sitosterol, kaempferol |
| Gamma-aminobutyric-acid receptor alpha-2 subunit | GABRA2 | beta-sitosterol, kaempferol |
| Muscarinic acetylcholine receptor M2 | CHRM2 | beta-sitosterol, kaempferol |
| Alpha-1B adrenergic receptor | ADRA1B | beta-sitosterol, kaempferol |
| Gamma-aminobutyric acid receptor subunit alpha-1 | GABRA1 | beta-sitosterol, kaempferol, paeoniflorigenone |
| Apoptosis regulator Bcl-2 | BCL2 | beta-sitosterol, kaempferol |
| Apoptosis regulator BAX | BAX | beta-sitosterol, kaempferol |
| Transcription factor AP-1 | JUN | beta-sitosterol, kaempferol |
| Caspase-3 | CASP3 | beta-sitosterol, kaempferol |
| Tumor necrosis factor | TNF | paeoniflorin, kaempferol |
| Transcription factor p65 | RELA | taxifolin, kaempferol |
| Mineralocorticoid receptor | NR3C2 | sitosterol, (3S,5R,8R,9R,10S,14S)-3,17-dihydroxy-4,4,8,10,14-pentamethyl-2,3,5,6,7,9-hexahydro-1H-cyclopenta[a]phenanthrene-15,16-dione |
| Nitric oxide synthase, inducible | NOS2 | peroxyergosterol, kaempferol |
| Intercellular adhesion molecule 1 | ICAM1 | taxifolin, kaempferol |
| Carbonic anhydrase IX | CA9 | 3,4-dihydroxybenzoicacid, kaempferol, phenol |
| Carbonic anhydrase VII | CA7 | 3,4-dihydroxybenzoicacid, kaempferol |
| \| Carbonic anhydrase IV \| \| --- \| | CA4 | 3,4-dihydroxybenzoicacid, kaempferol, phenol |
| Carbonic anhydrase II | CA2 | 3,4-dihydroxybenzoicacid, kaempferol, phenol, coumarinic acid |
| Carbonic anhydrase XII | CA12 | 3,4-dihydroxybenzoicacid, phenol |
| Carbonic anhydrase I | CA1 | 3,4-dihydroxybenzoicacid, coumarinic acid |
| Potassium voltage-gated channel subfamily H member 2 | KCNH2 | beta-sitosterol |
| Dopamine D1 receptor | DRD1 | beta-sitosterol |
| Muscarinic acetylcholine receptor M3 | CHRM3 | beta-sitosterol |
| Sodium channel protein type 5 subunit alpha | SCN5A | beta-sitosterol |
| Muscarinic acetylcholine receptor M4 | CHRM4 | beta-sitosterol |
| CGMP-inhibited 3',5'-cyclic phosphodiesterase A | PDE3A | beta-sitosterol |
| 5-hydroxytryptamine 2A receptor | HTR2A | beta-sitosterol |
| Gamma-aminobutyric-acid receptor alpha-5 subunit | GABRA5 | beta-sitosterol |
| Alpha-1A adrenergic receptor | ADRA1A | beta-sitosterol |
| Gamma-aminobutyric-acid receptor alpha-3 subunit | GABRA3 | beta-sitosterol |
| Beta-2 adrenergic receptor | ADRB2 | beta-sitosterol |
| Neuronal acetylcholine receptor subunit alpha-2 | CHRNA2 | beta-sitosterol |
| Sodium-dependent serotonin transporter | SLC6A4 | beta-sitosterol |
| Mu-type opioid receptor | OPRM1 | beta-sitosterol |
| Neuronal acetylcholine receptor protein, alpha-7 chain | CHRNA7 | beta-sitosterol |
| Caspase-9 | CASP9 | beta-sitosterol |
| Caspase-8 | CASP8 | beta-sitosterol |
| Protein kinase C alpha type | PRKCA | beta-sitosterol |
| Serum paraoxonase/arylesterase 1 | PON1 | beta-sitosterol |
| Microtubule-associated protein 2 | MAP2 | beta-sitosterol |
| Niemann-Pick C1-like protein 1 | NPC1L1 | beta-sitosterol |
| LXR-alpha | NR1H3 | beta-sitosterol |
| Nuclear receptor ROR-gamma | RORC | beta-sitosterol |
| HMG-CoA reductase | HMGCR | beta-sitosterol |
| Transforming growth factor beta-1 | TGFR1 | beta-sitosterol |
| Retinoic acid receptor RXR-alpha | RXRA | beta-sitosterol |
| Aldose reductase | AKR1B1 | beta-sitosterol |
| Diacylglycerol O-acyltransferase 2 | DGAT2 | beta-sitosterol |
| Microsomal triglyceride transfer protein large subunit | MTTP | beta-sitosterol |
| Apolipoprotein B-100 | APOB | beta-sitosterol |
| Carbonic anhydrase VI | CA6 | beta-sitosterol |
| Carbonic anhydrase XIV | CA14 | beta-sitosterol |
| Transient receptor potential cation channel subfamily A member 1 | TRPA1 | beta-sitosterol |
| Hydroxycarboxylic acid receptor 2 | HCAR2 | beta-sitosterol |
| Interleukin-6 | IL6 | beta-sitosterol |
| Monocyte differentiation antigen CD14 | CD14 | beta-sitosterol |
| Lipopolysaccharide-binding protein | LBP | beta-sitosterol |
| SUMO-activating enzyme | SAE1 | Mairin |
| DNA polymerase beta | POLB | Mairin |
| Aldo-keto reductase family 1 member B10 | AKR1B10 | Mairin |
| Protein-tyrosine phosphatase 1B | PTPN1 | Mairin |
| Androgen receptor | AR | kaempferol |
| Cell division control protein 2 homolog | CDK1 | kaempferol |
| Peroxisome proliferator activated receptor gamma | PPARG | kaempferol |
| Dipeptidyl peptidase IV | DPP4 | kaempferol |
| Trypsin-1 | PRSS1 | kaempferol |
| Nitric-oxide synthase, endothelial | NOS3 | kaempferol |
| Acetylcholinesterase | ACHE | kaempferol |
| Sodium-dependent noradrenaline transporter | SLC6A2 | kaempferol |
| Coagulation factor VII | F7 | kaempferol |
| Inhibitor of nuclear factor kappa-B kinase subunit beta | IKBKB | kaempferol |
| RAC-alpha serine/threonine-protein kinase | AKT1 | kaempferol |
| Activator of 90 kDa heat shock protein ATPase homolog 1 | AHSA1 | kaempferol |
| Mitogen-activated protein kinase 8 | MAPK8 | kaempferol |
| Interstitial collagenase | MMP1 | kaempferol |
| Signal transducer and activator of transcription 1-alpha/beta | STAT1 | kaempferol |
| Heme oxygenase 1 | HMOX1 | kaempferol |
| Cytochrome P450 3A4 | CYP3A4 | kaempferol |
| Cytochrome P450 1A2 | CYP1A2 | kaempferol |
| Cytochrome P450 1A1 | CYP1A1 | kaempferol |
| E-selectin | SELE | kaempferol |
| Vascular cell adhesion protein 1 | VCAM1 | kaempferol |
| Nuclear receptor subfamily 1 group I member 2 | NR1I2 | kaempferol |
| Cytochrome P450 1B1 | CYP1B1 | kaempferol |
| Arachidonate 5-lipoxygenase | ALOX5 | kaempferol |
| Hyaluronan synthase 2 | HAS2 | kaempferol |
| Glutathione S-transferase P | GSTP1 | kaempferol |
| Aryl hydrocarbon receptor | AHR | kaempferol |
| 26S proteasome non-ATPase regulatory subunit 3 | PSMD3 | kaempferol |
| Solute carrier family 2, facilitated glucose transporter member 4 | SLC2A4 | kaempferol |
| Nuclear receptor subfamily 1 group I member | NR1I3 | kaempferol |
| Insulin receptor | INSR | kaempferol |
| Type I iodothyronine deiodinase | DIO1 | kaempferol |
| Serine/threonine-protein phosphatase 2B catalytic subunit alpha isoform | PPP3CA | kaempferol |
| Glutathione S-transferase Mu 1 | GSTM1 | kaempferol |
| Glutathione S-transferase Mu 2 | GSTM2 | kaempferol |
| Aldo-keto reductase family 1 member C3 | AKR1C3 | kaempferol |
| Antileukoproteinase | SLPI | kaempferol |
| NADPH oxidase 4 | NOX4 | kaempferol |
| Xanthine dehydrogenase | XDH | kaempferol |
| Tyrosinase | TYR | kaempferol |
| Tyrosine-protein kinase receptor FLT3 | FLT3 | kaempferol |
| Estradiol 17-beta-dehydrogenase 2 | HSD17B2 | kaempferol |
| Multidrug resistance-associated protein 1 | ABCC1 | kaempferol |
| Estradiol 17-beta-dehydrogenase 1 | HSD17B1 | kaempferol |
| Estrogen-related receptor alpha | ESRRA | kaempferol |
| P-glycoprotein 1 | ABSB1 | kaempferol |
| ATP-binding cassette sub-family G member 2 | ABCG2 | kaempferol |
| Monoamine oxidase A | MAOA | kaempferol |
| Glyoxalase I | GLO1 | kaempferol |
| Tyrosine-protein kinase SYK | SYK | kaempferol |
| Glycogen synthase kinase-3 beta | GSK3B | kaempferol |
| Matrix metalloproteinase 9 | MMP9 | kaempferol |
| Matrix metalloproteinase 2 | MMP2 | kaempferol |
| Arachidonate 15-lipoxygenase | ALOX15 | kaempferol |
| Arachidonate 12-lipoxygenase | ALOX12B | kaempferol |
| Receptor-type tyrosine-protein phosphatase S | PTPRS | kaempferol |
| Adenosine A2a receptor | ADORA2A | kaempferol |
| Cyclin-dependent kinase 5/CDK5 activator 1 | CDK5R1 | kaempferol |
| G-protein coupled receptor 35 | GPR35 | kaempferol |
| Estrogen receptor beta | ESR2 | kaempferol |
| Death-associated protein kinase 1 | DAPK1 | kaempferol |
| DNA-3-methyladenine glycosylase | MPG | kaempferol |
| Solute carrier family 22 member 12 | SLC22A2 | kaempferol |
| Carbonic anhydrase III | CA3 | phenol |
