# Supplementary material for: A network pharmacology approach to predict potential targets and mechanisms of “Ramulus Cinnamomi (cassiae) – Paeonia lactiflora” herb pair in the treatment of chronic pain with comorbid anxiety and depression
Source: Ann Med. 2022 Jan 31;54(1):413–25. doi: 10.1080/07853890.2022.2031268 (PMC8812742; doi:10.1080/07853890.2022.2031268)
Supplement: Supplemental Material [file IANN_A_2031268_SM8833.zip › Supplemental files/Table S3.docx]

**Supplementary Table S3 The Top 10 targets of the PPI Network**

| Disease | Target | Degree | Betweenness Centrality | Closeness Centrality | Number of Directed Edges |
| --- | --- | --- | --- | --- | --- |
| CP | AKT1 | 62 | 0.16337307 | 0.66477273 | 62 |
|  | IL6 | 59 | 0.11245748 | 0.65 | 59 |
|  | TNF | 49 | 0.0570538 | 0.61256545 | 49 |
|  | PTGS2 | 43 | 0.05690278 | 0.56796117 | 43 |
|  | JUN | 43 | 0.04399126 | 0.57352941 | 43 |
|  | CASP3 | 42 | 0.03698294 | 0.57352941 | 42 |
|  | MAPK8 | 39 | 0.02459464 | 0.56521739 | 39 |
|  | PPARγ | 37 | 0.03335252 | 0.55188679 | 37 |
|  | NOS3 | 35 | 0.03534193 | 0.55714286 | 35 |
|  | MMP9 | 32 | 0.0247895 | 0.52232143 | 32 |
| AD | IL6 | 44 | 0.1292218 | 0.68571429 | 44 |
|  | AKT1 | 43 | 0.14340957 | 0.67924528 | 43 |
|  | TNF | 37 | 0.05088269 | 0.63157895 | 37 |
|  | CASP3 | 32 | 0.02852666 | 0.58064516 | 32 |
|  | JUN | 31 | 0.02944038 | 0.576 | 31 |
|  | PTGS2 | 30 | 0.01771767 | 0.56692913 | 30 |
|  | MAPK8 | 29 | 0.02452195 | 0.58064516 | 29 |
|  | NOS3 | 29 | 0.04238203 | 0.58064516 | 29 |
|  | PPARG | 28 | 0.0166697 | 0.55384615 | 28 |
|  | ICAM1 | 26 | 0.00742441 | 0.54135338 | 26 |
| MD | AKT1 | 36 | 0.13868596 | 0.6744186 | 36 |
|  | IL6 | 33 | 0.08857734 | 0.66666667 | 33 |
|  | TNF | 27 | 0.03152246 | 0.60416667 | 27 |
|  | CASP3 | 26 | 0.03241773 | 0.59183673 | 26 |
|  | JUN | 25 | 0.02909024 | 0.55769231 | 25 |
|  | NOS3 | 25 | 0.04374168 | 0.59183673 | 25 |
|  | PPARG | 24 | 0.01904454 | 0.55769231 | 24 |
|  | PTGS2 | 23 | 0.01422123 | 0.55769231 | 23 |
|  | MAPK8 | 23 | 0.01798526 | 0.56862745 | 23 |
|  | HMOX1 | 20 | 0.01303966 | 0.51785714 | 20 |

CP, chronic pain; AD, anxiety disorder; MD, mental depression
