# Supplementary material for: A network pharmacology approach to predict potential targets and mechanisms of “Ramulus Cinnamomi (cassiae) – Paeonia lactiflora” herb pair in the treatment of chronic pain with comorbid anxiety and depression
Source: Ann Med. 2022 Jan 31;54(1):413–25. doi: 10.1080/07853890.2022.2031268 (PMC8812742; doi:10.1080/07853890.2022.2031268)
Supplement: Supplemental Material [file IANN_A_2031268_SM8833.zip › Supplemental files/Table S4.docx]

**Supplementary Table S4 Pivotal results of GO Enrichment analysis for “Gui Zhi-Shao Yao” herb pair and CP**

| Category | Description | Enrichment | Z-score | Hits |
| --- | --- | --- | --- | --- |
| Biological Processes | hepoxilin metabolic process | 149.3013 | 29.80044 | ALOX5, ALOX12B, ALOX15, GSTM1, GSTM2, GSTP1 |
| Biological Processes | hepoxilin biosynthetic process | 149.3013 | 29.80044 | ALOX5, ALOX12B, ALOX15, GSTM1, GSTM2, GSTP1 |
| Biological Processes | adenylate cyclase-inhibiting G protein-coupled acetylcholine receptor signaling pathway | 111.976 | 21.02436 | CHRM1, CHRM2, CHRM3, OPRM1 |
| Biological Processes | regulation of cellular pH reduction | 95.97943 | 16.83137 | BCL2, CA2, CA7 |
| Biological Processes | regulation of systemic arterial blood pressure by norepinephrine-epinephrine | 74.65067 | 14.79975 | ADRA1B, ADRA1A, ADRB2 |
| Biological Processes | polyketide metabolic process | 74.65067 | 14.79975 | AKR1B1, AKR1C3, AKR1B10 |
| Biological Processes | daunorubicin metabolic process | 74.65067 | 14.79975 | AKR1B1, AKR1C3, AKR1B10 |
| Biological Processes | doxorubicin metabolic process | 74.65067 | 14.79975 | AKR1B1, AKR1C3, AKR1B10 |
| Biological Processes | lipoxygenase pathway | 69.985 | 18.48525 | ALOX5, ALOX12B, ALOX15, PON1, PTGS2 |
| Biological Processes | positive regulation of synaptic transmission, GABAergic | 68.90831 | 16.40144 | ADORA2A, ADRA1A, CA2, CA7 |
| Cellular Components | GABA-A receptor complex | 47.14779 | 13.47602 | GABRA1, GABRA2, GABRA3, GABRA5 |
| Cellular Components | GABA receptor complex | 44.7904 | 13.12006 | GABRA1, GABRA2, GABRA3, GABRA5 |
| Cellular Components | integral component of presynaptic membrane | 33.29016 | 18.62719 | ADORA2A, ADRA1A, CHRM1, CHRM2, CHRM3, DRD1, GABRA5, HTR2A, OPRM1, PTPRS, SLC6A4 |
| Cellular Components | dendrite membrane | 31.24912 | 13.29436 | GABRA1, GABRA2, GABRA3, GABRA5, INSR, OPRM1 |
| Cellular Components | intrinsic component of presynaptic membrane | 29.68039 | 17.52489 | ADORA2A, ADRA1A, CHRM1, CHRM2, CHRM3, DRD1, GABRA5, HTR2A, OPRM1, PTPRS, SLC6A4 |
| Cellular Components | neuron projection membrane | 25.69941 | 12.93337 | ADORA2A, GABRA1, GABRA2, GABRA3, GABRA5, INSR, OPRM1 |
| Cellular Components | integral component of postsynaptic membrane | 24.88356 | 17.33756 | ADORA2A, ADRA1A, CHRM1, CHRM2, CHRM3, DRD1, GABRA1, GABRA3, GABRA5, HTR2A, OPRM1, PTPRS, SLC6A4 |
| Cellular Components | caveola | 24.28395 | 14.22745 | ADRA1B, ADRA1A, HMOX1, HTR2A, INSR, NOS3, PTGS2, SCN5A, SELE |
| Cellular Components | neuronal cell body membrane | 23.99486 | 8.152928 | GABRA5, INSR, SLC6A2 |
| Cellular Components | intrinsic component of postsynaptic membrane | 23.86374 | 16.9497 | ADORA2A, ADRA1A, CHRM1, CHRM2, CHRM3, DRD1, GABRA1, GABRA3, GABRA5, HTR2A, OPRM1, PTPRS, SLC6A4 |
| Molecular Functions | estrogen 2-hydroxylase activity | 134.3712 | 19.97434 | CYP1A1, CYP1A2, CYP3A4 |
| Molecular Functions | carbonate dehydratase activity | 111.976 | 29.73718 | CA1, CA2, CA3, CA4, CA6, CA7, CA9, CA14 |
| Molecular Functions | arylesterase activity | 111.976 | 18.20697 | CA1, CA2, PON1 |
| Molecular Function | estrogen 16-alpha-hydroxylase activity | 111.976 | 21.02436 | CYP1A1, CYP1A2, CYP1B1, CYP3A4 |
| Molecular Functions | RNA polymerase II transcription coactivator binding | 111.976 | 18.20697 | AR, PGR, RELA |
| Molecular Functions | xenobiotic transmembrane transporting ATPase activity | 111.976 | 18.20697 | ABCC1, ABCB1, ABCG2 |
| Molecular Functions | G protein-coupled acetylcholine receptor activity | 95.97943 | 16.83137 | CHRM1, CHRM2, CHRM3 |
| Molecular Functions | RNA polymerase II transcription cofactor binding | 83.982 | 15.7209 | AR, PGR, RELA |
| Molecular Functions | retinal dehydrogenase activity | 83.982 | 15.7209 | AKR1B1, AKR1C3, AKR1B10 |
| Molecular Functions | benzodiazepine receptor activity | 81.43709 | 17.87 | GABRA1, GABRA2, GABRA3, GABRA5 |

GO, Gene Ontology; CP, chronic pain.
