# Supplementary material for: A network pharmacology approach to predict potential targets and mechanisms of “Ramulus Cinnamomi (cassiae) – Paeonia lactiflora” herb pair in the treatment of chronic pain with comorbid anxiety and depression
Source: Ann Med. 2022 Jan 31;54(1):413–25. doi: 10.1080/07853890.2022.2031268 (PMC8812742; doi:10.1080/07853890.2022.2031268)
Supplement: Supplemental Material [file IANN_A_2031268_SM8833.zip › Supplemental files/Table S5.docx]

**Supplementary Table S5 Pivotal results of GO Enrichment analysis for “Gui Zhi-Shao Yao” herb pair and AD**

| Category | Description | Enrichment | Z-score | Hits |
| --- | --- | --- | --- | --- |
| Biological Processes | adenylate cyclase-inhibiting G protein-coupled acetylcholine receptor signaling pathway | 191.739726 | 27.58902806 | CHRM1, CHRM2, CHRM3, OPRM1 |
| Biological Processes | hepoxilin metabolic process | 127.826484 | 19.4575977 | ALOX5, GSTM1, GSTP1 |
| Biological Processes | hepoxilin biosynthetic process | 127.826484 | 19.4575977 | ALOX5, GSTM1, GSTP1 |
| Biological Processes | positive regulation of leukocyte adhesion to vascular endothelial cell | 100.0381179 | 24.29589868 | ALOX5, ICAM1, IL6, RELA, SELE, TNF |
| Biological Processes | positive regulation of synaptic transmission, GABAergic | 88.49525817 | 16.1341186 | ADORA2A, ADRA1A, CA2 |
| Biological Processes | negative regulation of production of miRNAs involved in gene silencing by miRNA | 82.1741683 | 15.53383466 | IL6, TGFB1, TNF |
| Biological Processes | G protein-coupled acetylcholine receptor signaling pathway | 80.73251622 | 17.77656686 | CHRM1, CHRM2, CHRM3, OPRM1 |
| Biological Processes | long-chain fatty acid biosynthetic process | 78.95165189 | 23.25502794 | ALOX5, CYP1A1, CYP1A2, CYP3A4, GSTM1, GSTP1, PTGS2 |
| Biological Processes | positive regulation of macrophage differentiation | 76.69589041 | 14.99417258 | CASP8, PRKCA, TGFB1 |
| Biological Processes | positive regulation of amyloid-beta formation | 76.69589041 | 17.31532639 | CASP3, CHRNA7, RELA, TNF |
| Cellular Components | GABA-A receptor complex | 80.73251622 | 17.77656686 | GABRA1, GABRA2, GABRA3, GABRA5 |
| Cellular Components | GABA receptor complex | 76.69589041 | 17.31532639 | GABRA1, GABRA2, GABRA3, GABRA5 |
| Cellular Components | dendrite membrane | 53.50876075 | 17.61927318 | GABRA1, GABRA2, GABRA3, GABRA5, INSR, OPRM1 |
| Cellular Components | integral component of presynaptic membrane | 51.82154757 | 22.38343353 | ADORA2A, ADRA1A, CHRM1, CHRM2, CHRM3, DRD1, GABRA5, HTR2A, OPRM1, SLC6A4 |
| Cellular Components | intrinsic component of presynaptic membrane | 46.20234362 | 21.08788226 | ADORA2A, ADRA1A, CHRM1, CHRM2, CHRM3, DRD1, GABRA5, HTR2A, OPRM1, SLC6A4 |
| Cellular Components | neuron projection membrane | 44.00583876 | 17.1931011 | ADORA2A, GABRA1, GABRA2, GABRA3, GABRA5, INSR, OPRM1 |
| Cellular Components | neuronal cell body membrane | 41.08708415 | 10.85148001 | GABRA5, INSR, SLC6A2 |
| Cellular Components | integral component of postsynaptic membrane | 39.33122585 | 21.24434226 | ADORA2A, ADRA1A, CHRM1, CHRM2, CHRM3, DRD1, GABRA1, GABRA3, GABRA5, HTR2A, OPRM1, SLC6A4 |
| Cellular Components | intrinsic component of postsynaptic membrane | 37.71929037 | 20.78312243 | ADORA2A, ADRA1A, CHRM1, CHRM2, CHRM3, DRD1, GABRA1, GABRA3, GABRA5, HTR2A, OPRM1, SLC6A4 |
| Cellular Components | cell body membrane | 37.11091471 | 10.28603797 | GABRA5, INSR, SLC6A2 |
| Molecular Functions | estrogen 2-hydroxylase activity | 230.0876712 | 26.19471613 | CYP1A1, CYP1A2, CYP3A4 |
| Molecular Functions | G protein-coupled acetylcholine receptor activity | 164.3483366 | 22.10071022 | CHRM1, CHRM2, CHRM3 |
| Molecular Functions | estrogen 16-alpha-hydroxylase activity | 143.8047945 | 20.65561048 | CYP1A1, CYP1A2, CYP3A4 |
| Molecular Functions | benzodiazepine receptor activity | 139.4470735 | 23.48300411 | GABRA1, GABRA2, GABRA3, GABRA5 |
| Molecular Functions | GABA-gated chloride ion channel activity | 117.9936776 | 21.57362478 | GABRA1, GABRA2, GABRA3, GABRA5 |
| Molecular Functions | acetylcholine binding | 117.9936776 | 21.57362478 | ACHE, CHRM3, CHRNA2, CHRNA7 |
| Molecular Functions | oxidoreductase activity, acting on CH or CH2 groups | 115.0438356 | 18.44325569 | CYP1A2, CYP3A4, XDH |
| Molecular Functions | inhibitory extracellular ligand-gated ion channel activity | 102.2611872 | 20.05825823 | GABRA1, GABRA2, GABRA3, GABRA5 |
| Molecular Functions | acetylcholine receptor activity | 87.15442092 | 20.67034932 | CHRM1, CHRM2, CHRM3, CHRNA2, CHRNA7 |
| Molecular Functions | GABA-A receptor activity | 80.73251622 | 17.77656686 | GABRA1, GABRA2, GABRA3, GABRA5 |

GO, Gene Ontology; AD, anxiety disorder.
