# Supplementary material for: A network pharmacology approach to predict potential targets and mechanisms of “Ramulus Cinnamomi (cassiae) – Paeonia lactiflora” herb pair in the treatment of chronic pain with comorbid anxiety and depression
Source: Ann Med. 2022 Jan 31;54(1):413–25. doi: 10.1080/07853890.2022.2031268 (PMC8812742; doi:10.1080/07853890.2022.2031268)
Supplement: Supplemental Material [file IANN_A_2031268_SM8833.zip › Supplemental files/Table S6.docx]

**Supplementary Table S6 Pivotal results of GO Enrichment analysis for “Gui Zhi-Shao Yao” herb pair and MD**

| Category | Description | Enrichment | Z-score | Hits |
| --- | --- | --- | --- | --- |
| Biological Processes | negative regulation of production of miRNAs involved in gene silencing by miRNA | 135.5641646 | 23.14440245 | IL6, PPP3CA, TGFB1, TNF |
| Biological Processes | positive regulation of synaptic transmission, GABAergic | 109.494133 | 17.98136696 | ADORA2A, ADRA1A, CA2 |
| Biological Processes | negative regulation of gene silencing by RNA | 103.146647 | 22.52218009 | IL6, PPARG, PPP3CA, TGFB1, TNF |
| Biological Processes | regulation of chemokine biosynthetic process | 94.89491525 | 16.71660189 | HMOX1, IL6, TNF |
| Biological Processes | regulation of synaptic transmission, dopaminergic | 88.96398305 | 16.17457711 | DRD1, PTGS2, SLC6A4 |
| Biological Processes | negative regulation of lipid storage | 86.26810478 | 18.38707218 | IL6, PPARG, TNF, NR1H3 |
| Biological Processes | regulation of extracellular matrix disassembly | 83.73080758 | 15.68077571 | DPP4, IL6, TGFB1 |
| Biological Processes | heat generation | 83.73080758 | 15.68077571 | ADRB2, PTGS2, TNF |
| Biological Processes | regulation of cholesterol storage | 79.07909605 | 15.22841171 | APOB, PPARG, NR1H3 |
| Biological Processes | cellular response to cadmium ion | 74.91703836 | 20.95440001 | AKT1, CYP1A2, HMOX1, JUN, MMP9, MAPK8 |
| Cellular Components | GABA-A receptor complex | 99.88938448 | 19.81608318 | GABRA1, GABRA2, GABRA3, GABRA5 |
| Cellular Components | GABA receptor complex | 94.89491525 | 19.30439418 | GABRA1, GABRA2, GABRA3, GABRA5 |
| Cellular Components | dendrite membrane | 66.20575483 | 19.66517744 | GABRA1, GABRA2, GABRA3, GABRA5, INSR, OPRM1 |
| Cellular Components | neuron projection membrane | 54.4479022 | 19.20495 | ADORA2A, GABRA1, GABRA2, GABRA3, GABRA5, INSR, OPRM1 |
| Cellular Components | integral component of presynaptic membrane | 51.29454879 | 19.90927563 | ADORA2A, ADRA1A, CHRM2, DRD1, GABRA5, HTR2A, OPRM1, SLC6A4 |
| Cellular Components | neuronal cell body membrane | 50.83656174 | 12.12518299 | GABRA5, INSR, SLC6A2 |
| Cellular Components | cell body membrane | 45.91689448 | 11.49939726 | GABRA5, INSR, SLC6A2 |
| Cellular Components | intrinsic component of presynaptic membrane | 45.73248928 | 18.75645694 | ADORA2A, ADRA1A, CHRM2, DRD1, GABRA5, HTR2A, OPRM1, SLC6A4 |
| Cellular Components | integral component of postsynaptic membrane | 40.55338259 | 19.70289003 | ADORA2A, ADRA1A, CHRM2, DRD1, GABRA1, GABRA3, GABRA5, HTR2A, OPRM1, SLC6A4 |
| Cellular Components | caveola | 40.01592812 | 16.35948698 | ADRA1A, HMOX1, HTR2A, INSR, NOS3, PTGS2, SCN5A |
| Molecular Functions | estrogen 16-alpha-hydroxylase activity | 177.9279661 | 23.00103897 | CYP1A2, CYP1B1, CYP3A4 |
| Molecular Functions | benzodiazepine receptor activity | 172.5362096 | 26.15058482 | GABRA1, GABRA2, GABRA3, GABRA5 |
| Molecular Functions | GABA-gated chloride ion channel activity | 145.9921773 | 24.03040122 | GABRA1, GABRA2, GABRA3, GABRA5 |
| Molecular Functions | inhibitory extracellular ligand-gated ion channel activity | 126.5265537 | 22.34814759 | GABRA1, GABRA2, GABRA3, GABRA5 |
| Molecular Functions | acetylcholine binding | 109.494133 | 17.98136696 | ACHE, CHRNA2, CHRNA7 |
| Molecular Functions | GABA-A receptor activity | 99.88938448 | 19.81608318 | GABRA1, GABRA2, GABRA3, GABRA5 |
| Molecular Functions | ligand-gated anion channel activity | 99.88938448 | 19.81608318 | GABRA1, GABRA2, GABRA3, GABRA5 |
| Molecular Functions | GABA receptor activity | 86.26810478 | 18.38707218 | GABRA1, GABRA2, GABRA3, GABRA5 |
| Molecular Functions | acetylcholine receptor activity | 64.70107858 | 13.73639455 | CHRM2, CHRNA2, CHRNA7 |
| Molecular Functions | transmitter-gated ion channel activity involved in regulation of postsynaptic membrane potential | 60.5712225 | 18.7842662 | CHRNA2, CHRNA7, GABRA1, GABRA2, GABRA3, GABRA5 |

GO, Gene Ontology; MD, mental depression.
