# Supplementary material for: A network pharmacology approach to predict potential targets and mechanisms of “Ramulus Cinnamomi (cassiae) – Paeonia lactiflora” herb pair in the treatment of chronic pain with comorbid anxiety and depression
Source: Ann Med. 2022 Jan 31;54(1):413–25. doi: 10.1080/07853890.2022.2031268 (PMC8812742; doi:10.1080/07853890.2022.2031268)
Supplement: Supplemental Material [file IANN_A_2031268_SM8833.zip › Supplemental files/Table S7.docx]

**Supplementary Table S7 The KEGG Pathway Enrichment Analysis (CP)**

| Pathway | Enrichment | -Log*P* | Hits |
| --- | --- | --- | --- |
| hsa00910: Nitrogen metabolism | 99.534 | 14.275 | CA1, CA2, CA3, CA4, CA6, CA7, CA9, CA14 |
| hsa00232: Caffeine metabolism | 67.186 | 4.992 | CYP1A2, SLC6A2, XDH |
| hsa01523: Antifolate resistance | 43.346 | 8.326 | IKBKB, IL6, ABCC1, RELA, TNF, ABCG2 |
| hsa04215: Apoptosis - multiple species | 40.719 | 8.152 | BAX, BCL2, CASP3, CASP8, CASP9, MAPK8 |
| hsa04933: AGE-RAGE signaling pathway in diabetic complications | 37.674 | 22.935 | AKT1, BAX, BCL2, CASP3, ICAM1, IL6, JUN, MMP2, NOS3, PRKCA, MAPK8, RELA, SELE, STAT1, TGFB1, TNF, VCAM1, NOX4 |
| hsa04657: IL-17 signaling pathway | 28.289 | 13.835 | CASP3, CASP8, GSK3B, IKBKB, IL6, JUN, MMP1, MMP9, MAPK8, PTGS2, RELA, TNF |
| hsa04668: TNF signaling pathway | 27.264 | 15.840 | AKT1, CASP3, CASP8, ICAM1, IKBKB, IL6, JUN, MMP9, MAPK8, PTGS2, RELA, SELE, TNF, VCAM1 |
| hsa05033: Nicotine addiction | 26.661 | 5.913 | CHRNA7, GABRA1, GABRA2, GABRA3, GABRA5 |
| hsa05418: Fluid shear stress and atherosclerosis | 25.724 | 18.725 | AKT1, BCL2, GSTM1, GSTM2, GSTP1, HMOX1, ICAM1, IKBKB, JUN, MMP2, MMP9, NOS3, MAPK8, RELA, SELE, TNF, VCAM1 |
| hsa04625: c-type lectin receptor signaling pathway | 25.116 | 13.197 | AKT1, CASP8, IKBKB, IL6, JUN, PPP3CA, MAPK8, PTGS2, RELA, STAT1, SYK, TNF |
| hsa05030: Cocaine addiction | 24.431 | 6.769 | DRD1, JUN, MAOA, PRKCA, RELA, CDK5R1 |
| hsa00790: Folate biosynthesis | 23.995 | 3.581 | AKR1B1, AKR1C3, AKR1B10 |
| hsa04931: insulin resistance | 23.783 | 12.908 | AKT1, GSK3B, IKBKB, IL6, INSR, NOS3, MAPK8, PTPN1, RELA, SLC2A4, TNF, NR1H3 |
| hsa05014: Amyotrophic lateral sclerosis | 23.574 | 6.675 | BAX, BCL2, CASP3, CASP9, PPP3CA, TNF |
| hsa05321: Inflammatory bowel disease | 23.398 | 7.682 | IL6, JUN, RELA, RORC, STAT1, TGFB1, TNF |
| hsa04923: Regulation of lipolysis in adipocytes | 23.167 | 3.535 | ALOX15, CYP1A2, CYP3A4 |
| hsa00591: Linoleic acid metabolism | 23.167 | 6.629 | ADRB2, AKT1, INSR, PRKCA, PTGS1, PTGS2 |
| hsa04620: Toll-like receptor signaling pathway | 22.193 | 11.535 | AKT1, CASP8, CD14, IKBKB, IL6, JUN, LBP, MAPK8, RELA, STAT1, TNF |
| hsa00140: Steroid hormone biosynthesis | 22.028 | 6.496 | CYP1A1, CYP1A2, CYP1B1, CYP3A4, HSD17B1, AKR1C3 |
| hsa00590: Arachidonic acid metabolism | 21.329 | 6.412 | ALOX5, ALOX12B, ALOX15, PTGS1, PTGS2, AKR1C3 |

KEGG, Kyoto Encyclopedia of Genes and Genomes; CP, chronic pain.
