# Supplementary material for: A network pharmacology approach to predict potential targets and mechanisms of “Ramulus Cinnamomi (cassiae) – Paeonia lactiflora” herb pair in the treatment of chronic pain with comorbid anxiety and depression
Source: Ann Med. 2022 Jan 31;54(1):413–25. doi: 10.1080/07853890.2022.2031268 (PMC8812742; doi:10.1080/07853890.2022.2031268)
Supplement: Supplemental Material [file IANN_A_2031268_SM8833.zip › Supplemental files/Table S8.docx]

**Supplementary Table S8 The KEGG Pathway Enrichment Analysis (AD)**

| Pathway | Enrichment | -Log*P* | Hits |
| --- | --- | --- | --- |
| hsa00232: Caffeine metabolism | 115.0438356 | 5.695759023 | CYP1A2, SLC6A2, XDH |
| hsa04933: AGE-RAGE signaling pathway in diabetic complications | 57.3427218 | 23.54746956 | AKT1, BCL2, CASP3, ICAM1, IL6, JUN, MMP2, NOS3, PRKCA, MAPK8, RELA, SELE, STAT1, TGFB1, TNF, VCAM1 |
| hsa01523: Antifolate resistance | 49.48121962 | 5.89645928 | IKBKB, IL6, RELA, TNF |
| hsa04657: IL-17 signaling pathway | 48.43950973 | 16.7539552 | CASP3, CASP8, GSK3B, IKBKB, IL6, JUN, MMP1, MMP9, MAPK8, PTGS2, RELA, TNF |
| hsa04668: TNF signaling pathway | 46.68445503 | 19.2843146 | AKT1, CASP3, CASP8, ICAM1, IKBKB, IL6, JUN, MMP9, MAPK8, PTGS2, RELA, SELE, TNF, VCAM1 |
| hsa04215: Apoptosis - multiple species | 46.48235782 | 5.784063545 | BCL2, CASP3, CASP8, MAPK8 |
| hsa05033: Nicotine addiction | 45.65231572 | 7.082098594 | CHRNA7, GABRA1, GABRA2, GABRA3, GABRA5 |
| hsa05418: Fluid shear stress and atherosclerosis | 41.45723806 | 21.18036781 | AKT1, BCL2, GSTM1, GSTP1, HMOX1, ICAM1, IKBKB, JUN, MMP2, MMP9, NOS3, MAPK8, RELA, SELE, TNF, VCAM1 |
| hsa04923: Regulation of lipolysis in adipocytes | 39.67028814 | 8.032968741 | ADRB2, AKT1, INSR, PRKCA, PTGS1, PTGS2 |
| hsa04625: c-type lectin receptor signaling pathway | 39.42312124 | 14.3581284 | AKT1, CASP8, IKBKB, IL6, JUN, MAPK8, PTGS2, RELA, STAT1, SYK, TNF |
| hsa05030: Cocaine addiction | 34.86176837 | 6.481862461 | DRD1, JUN, MAOA, PRKCA, RELA |
| hsa05321: Inflammatory bowel disease | 34.34144347 | 7.649070545 | IL6, JUN, RELA, STAT1, TGFB1, TNF |
| hsa04931: insulin resistance | 33.9362347 | 12.41557435 | AKT1, GSK3B, IKBKB, IL6, INSR, NOS3, MAPK8, RELA, SLC2A4, TNF |
| hsa04930: Type II diabetes mellitus | 33.05857345 | 6.364857681 | IKBKB, INSR, MAPK8, SLC2A4, TNF |
| hsa04920: Adipocytokine signaling pathway | 31.956621 | 7.458849935 | AKT1, IKBKB, MAPK8, RELA, SLC2A4, TNF |
| hsa04620: Toll-like receptor signaling pathway | 31.09292854 | 10.86389452 | AKT1, CASP8, IKBKB, IL6, JUN, MAPK8, RELA, STAT1, TNF |
| hsa04926: relaxin signaling pathway | 31.01672039 | 13.18749848 | AKT1, JUN, MMP1, MMP2, MMP9, NOS2, NOS3, PRKCA, MAPK8, RELA, TGFB1 |
| hsa04917: Prolactin signaling pathway | 30.67835616 | 7.351340824 | AKT1, ESR2, GSK3B, MAPK8, RELA, STAT1 |
| hsa04725: Cholinergic synapse | 30.01143538 | 10.72394448 | ACHE, AKT1, BCL2, CHRM1, CHRM2, CHRM3, CHRNA7, PIK3CG, PRKCA |
| hsa04960: Aldosterone-regulated sodium reabsorption | 29.49841939 | 3.837637664 | INSR, NR3C2, PRKCA |

KEGG, Kyoto Encyclopedia of Genes and Genomes; AD, anxiety disorder.
