# Supplementary material for: A network pharmacology approach to predict potential targets and mechanisms of “Ramulus Cinnamomi (cassiae) – Paeonia lactiflora” herb pair in the treatment of chronic pain with comorbid anxiety and depression
Source: Ann Med. 2022 Jan 31;54(1):413–25. doi: 10.1080/07853890.2022.2031268 (PMC8812742; doi:10.1080/07853890.2022.2031268)
Supplement: Supplemental Material [file IANN_A_2031268_SM8833.zip › Supplemental files/Table S9.docx]

**Supplementary Table S9 The KEGG Pathway Enrichment Analysis (MD)**

| Pathway | Enrichment | -Log*P* | Hits |
| --- | --- | --- | --- |
| hsa04933: AGE-RAGE signaling pathway in diabetic complications | 62.08078568 | 21.16901505 | AKT1, BAX, CASP3, ICAM1, IL6, JUN, MMP2, NOS3, PRKCA, MAPK8, STAT1, TGFB1, TNF, VCAM1 |
| hsa05033: Nicotine addiction | 56.4850686 | 7.552552641 | CHRNA7, GABRA1, GABRA2, GABRA3, GABRA5 |
| hsa04215: Apoptosis - multiple species | 43.13405239 | 4.333662873 | BAX, CASP3, MAPK8 |
| hsa04668: TNF signaling pathway | 41.25865881 | 13.31236037 | AKT1, CASP3, ICAM1, IL6, JUN, MMP9, MAPK8, PTGS2, TNF, VCAM1 |
| hsa04923: Regulation of lipolysis in adipocytes | 40.90298071 | 6.83241972 | ADRB2, AKT1, INSR, PRKCA, PTGS2 |
| hsa04657: IL-17 signaling pathway | 39.95575379 | 10.60123289 | CASP3, GSK3B, IL6, JUN, MMP9, MAPK8, PTGS2, TNF |
| hsa05418: Fluid shear stress and atherosclerosis | 38.47091159 | 15.56228258 | AKT1, GSTM1, GSTP1, HMOX1, ICAM1, JUN, MMP2, MMP9, NOS3, MAPK8, TNF, VCAM1 |
| hsa04960: Aldosterone-regulated sodium reabsorption | 36.49804433 | 4.113542194 | INSR, NR3C2, PRKCA |
| hsa04370: VEGF signaling pathway | 36.49804433 | 6.581398933 | AKT1, NOS3, PPP3CA, PRKCA, PTGS2 |
| hsa05032: Morphine addiction | 36.10132646 | 9.011299972 | DRD1, GABRA1, GABRA2, GABRA3, GABRA5, OPRM1, PRKCA |
| hsa04625: c-type lectin receptor signaling pathway | 35.47473467 | 10.18129849 | AKT1, IL6, JUN, PPP3CA, MAPK8, PTGS2, STAT1, TNF |
| hsa05321: Inflammatory bowel disease | 35.40855047 | 6.514897983 | IL6, JUN, STAT1, TGFB1, TNF |
| hsa01522: Endocrine resistance | 34.59710452 | 8.880412581 | AKT1, BAX, ESR2, JUN, MMP2, MMP9, MAPK8 |
| hsa05030: Cocaine addiction | 34.50724191 | 5.251813188 | DRD1, JUN, MAOA, PRKCA |
| hsa00982: Drug metabolism - cytochrome P450 | 33.89104116 | 6.41897013 | CYP1A2, CYP3A4, GSTM1, GSTP1, MAOA |
| hsa04931: insulin resistance | 33.59112044 | 9.989646852 | AKT1, GSK3B, IL6, INSR, NOS3, MAPK8, TNF, NR1H3 |
| hsa05014: Amyotrophic lateral sclerosis | 33.29646149 | 5.189393427 | BAX, CASP3, PPP3CA, TNF |
| hsa04917: Prolactin signaling pathway | 31.63163842 | 6.268328505 | AKT1, ESR2, GSK3B, MAPK8, STAT1 |
| hsa04926: relaxin signaling pathway | 31.39905284 | 10.92827412 | AKT1, JUN, MMP2, MMP9, NOS2, NOS3, PRKCA, MAPK8, TGFB1 |
| hsa00380: Tryptophan metabolism | 28.46847458 | 3.789336994 | CYP1A2, CYP1B1, MAOA |

KEGG, Kyoto Encyclopedia of Genes and Genomes; MD, mental depression.
